# TRAJECTORIES OF INFORMAL AND FORMAL SOCIAL PARTICIPATION AFTER RETIREMENT

**DOI:** 10.1093/geroni/igac059.2498

**Published:** 2022-12-20

**Authors:** Jeremy Lim-Soh, Shannon Ang, Rahul Malhotra

**Affiliations:** Duke-NUS Medical School, National University of Singapore, Singapore, Singapore; Nanyang Technological University, Singapore, Singapore; Duke-NUS Medical School, Singapore, Singapore

## Abstract

Literature suggests that social participation, a component of successful ageing, declines on average after retirement, but to whom does this experience apply? We sought to identify contrasting longitudinal trajectories of social participation after retirement, and their associated individual-level correlates. Seven waves of the Korean Longitudinal Study of Ageing provided data on the informal and formal social participation, measured by frequencies of meeting a friend and attending a group respectively, of individuals 45 years and older who left work. Group-based trajectory modeling captured heterogenous changes over time in social participation after retirement. Multinomial logit regressions estimated individual-level correlates of the trajectories, including whether the individual returns to work. While a sizeable minority of respondents did experience decreasing trajectories of informal (17%) and formal (23%) participation, a majority exhibited stable trajectories of either type of social participation, and some experienced increasing formal (9%) participation. Employment type, age, gender, education, marital status, region, health, and economic satisfaction were associated with the trajectories. Returning to work, versus stopping work for an extended period, was associated with moderate stable or increasing trajectories of social participation. The findings challenge the belief that decline in social participation is the norm after retirement. They underscore the presence of heterogenous experiences of social participation after retirement, and identify vulnerable sub-groups that do experience decline. Furthermore, as returning to work may be beneficial for social participation, future studies should examine ways in which bridge employment can support successful aging.

